# Hepatitis E immunosuppressed patients and assisted pregnancy: Is it time to discuss neglected issues?

**Published:** 2017

**Authors:** D Anyfantakis, EK Symvoulakis, S Kastanakis

**Affiliations:** * Primary Health Care Centre of Kissamos, Chania, Crete, Greece; ** Private Family Practice Unit, Heraklion, Crete, Greece; *** Department of Internal Medicine, Saint George General Hospital of Chania, Crete, Greece

**To the Editor:**

Hepatitis E virus (HEV) represents the most important etiological factor of acute clinical hepatitis among adults in many regions of the developing world, with significant adverse outcomes in terms of morbidity and mortality related burden especially among pregnant women [**1**]. Remarkably, it has been reported that 2 out of 10 women with HEV may die during the third trimester [**2**]. Despite that, epidemiological data on infection and disease during childhood are limited [**2**]. HEV infection usually causes a mild self-limiting disease, although fulminant hepatitis and death are also described [**2**]. Liver transplant recipients, who developed a chronic HEV infection through blood transfusion, subsequently developed permanent hepatic graft damage [**3**]. A recent cohort study, by Zhang and colleagues, among more than 110.000 healthy participants of 16 to 65 years old in China [**4**] suggested that the infection with hepatitis E virus (HEV) genotype 4 could be a vaccine-preventable disease.

Currently, infection with hepatitis E has been reported to be a geographically widely distributed disease [**1**]. HEV is considered more prevalent in industrialized countries than previously thought, while HEV acquisition through blood transfusion is underestimated, causing persistent disease in immunosuppressed patients [**5**]. Currently, the reduction of the risk of HEV through blood donation screening has been controversial [**5**]. An interesting review in this direction, by Wang et al., suggested that immunization against HEV infection would be beneficial for populations at risk [**6**]. Furthermore, the immunological therapies during the last decades in general have been considered that will determine a promising role [**7**]. 

Accordingly, we advocate the need for cost-effectiveness studies in order to discuss a potential consensus to vaccinate a pool of blood donors, who donate haema products to immunosuppressed or undergoing transplantation patients and women receiving assisted conception treatments.
